# Caregiver Challenges of Older University Employees During the COVID-19 Pandemic

**DOI:** 10.1093/geroni/igab046.250

**Published:** 2021-12-17

**Authors:** Heather Kenning, Yunjia Yang, Lisa O'Neill, Mindy Fain, Mark Wager, Amanda Sokan

**Affiliations:** 1 University of Arizona, Tucson, Arizona, United States; 2 University of Arizona, Center on Aging, Tucson, Arizona, United States; 3 University of Arizona Center on Aging, Tucson, Arizona, United States; 4 University of Arizona, Phoenix, Arizona, United States

## Abstract

The COVID-19 pandemic has created numerous challenges for older employees who are also caregivers. Some challenges are associated with disruptions in community-based support services leading to the intensification of caregiver responsibilities. Other challenges are related to caregivers’ concerns about their health or the risk of bringing the virus to the care recipient. This study investigated the impacts of those challenges on older (age 55+) working caregivers, from a major university, with a sample that included 57 male and 80 female caregivers. The investigation explored the association of gender and perception of COVID risk, vulnerability, loneliness, resilience, and interpersonal change. Although literature suggests that female caregivers report more risk-perception, stress, and overburden than male caregivers, our findings showed no significant differences based on gender. These findings suggest the importance of understanding that both male and female older working caregivers have been affected by stress and overburden due to the recent pandemic.

